# Incidence, risk factors, and whole-genome sequence of SARs-CoV-2 and influenza virus among the Egyptian pilgrims returning from Umrah mass gathering in Saudi Arabia, April-May 2022

**DOI:** 10.1016/j.jiph.2022.10.005

**Published:** 2022-11

**Authors:** Amr Kandeel, Manal Fahim, Ola Deghedy, Walaa Alim, Wael H. Roshdy, Mohamed K. Khalifa, Rabeh El Shesheny, Ahmed Kandeil, Amel Naguib, Nancy Elguindy, Mohammad Abdel Fattah, Salma Afifi, Amira Mohsen, Khaled Abdelghaffar

**Affiliations:** a Preventive Sector, Ministry of Health and Population, Cairo, Egypt; b Department of Epidemiology and Surveillance, Preventive Sector, Ministry of Health and Population, Cairo, Egypt; c Central Public Health Laboratory, Ministry of Health and Population, Cairo, Egypt; d Centre of Scientific Excellence for Influenza Viruses, National Research Centre, 12622 Dokki, Giza, Egypt; e Ministry of Health and Population Consultant, Cairo, Egypt; f World Health Organization, Egypt Country Office, Cairo, Egypt; g Ministry of Health and Population, Cairo, Egypt

**Keywords:** COVID-19, SARS-CoV-2, Whole-genome sequencing, Influenza virus, Incidence, Umrah pilgrims, Egypt

## Abstract

**Background:**

Ramadan Umrah is the second largest Islamic pilgrimage with 2.75 million pilgrims allowed in 2022. This report presents the results of a survey among Egyptian pilgrims returning from Ramadan Umrah to monitor SARS-CoV-2 and influenza activity and identify prevalent SARS-CoV-2 variants after this mass gathering.

**Methods:**

Cross‐sectional survey conducted at Cairo airport from 30th April 2022–5 th May 2022. Pilgrims were invited to participate voluntarily. After consenting, participants interviewed using questionnaire including demographics, health status, and vaccination information and asked to provide NP/OP swabs for SARS-CoV-2 and influenza testing by RT-PCR. Whole-genome sequencing performed for 29 SARS-CoV-2 isolates. Incidence calculated, descriptive data analysis performed, and SARS-CoV-2 patients were compared to negatively tested participants using chi^2^ and p value< 0.05.

**Results:**

Overall, 1003 subjects participated, their mean age 50.9 ± 13 years, 594 (59.2%) were males. Of them, 76(7.6%) tested positive including 67(6.7%) SARS-CoV-2, 7(0.7%) influenza and 2(0.2%) SARS-CoV-2/influenza coinfection. Omicron sublineage BA.2 was the prevalent variant with no difference in severity identified between BA.1 and BA.2. No difference was identified between COVID-19 incidence among receivers of different vaccine types or between fully vaccinated and booster dose receivers.

**Conclusions:**

Survey indicated a low incidence of SARs-CoV-2 and influenza among Egyptian pilgrims returning from Ramadan Umrah. Patients had mild or no symptoms with no hospitalization or deaths reported. Full vaccination and booster doses of COVID-19 vaccines proved equally effective. Enhancing COVID-19 and influenza vaccination before mass gatherings and close monitoring of respiratory viruses among pilgrims returning from Hajj and Umrah are crucial for outbreak early detection and mitigation.

## 1 Introduction

The Hajj is the largest gathering of Muslims held each year in Mecca and Medina in Saudi Arabia. It is the largest religious gathering in the world with over 2 million attendees from more than 180 countries every year [1]. Hajj takes place between the 8th and the 13th of Dhul Hijjah, the 12th month of the Islamic calendar. Umrah is the second largest Islamic pilgrimage that can be undertaken at any time of the year. Umrah during Ramadan, the ninth month of the Islamic calendar holds the same religious value as Hajj [2]. As a result of the COVID-19 pandemic, Saudi Arabia has suspended Hajj and Umrah starting in March 2020.

Globally, over 468 million confirmed cases and over 6 million deaths have been reported globally by end of March 2022 [3]. Whereas 749,965 confirmed cases and 9033 deaths were reported from Saudi Arabia in the same period [4]. To reduce the extent of COVID-19 epidemic locally and globally, Hajj was restricted to pilgrims already in the country, excluding those with chronic diseases and those over 65 [5].

As the pandemic progressed towards endemicity, many countries including Saudi Arabia started to ease COVID-19 restrictions [6]. In April 2022, the Saudi Ministry of Hajj and Umrah lifted most COVID-19 restrictions on entry into Saudi Arabia for international pilgrims. Two years after pandemic restrictions drastically reduced pilgrimages, more than 2.75 million pilgrims undertook Ramadan Umrah in the year 2022, and one million will have the chance to make Hajj [7].

Egypt is one of the countries that send a large number of pilgrims to the Hajj every year [2]. Saudi authorities required all Egyptians traveling to Saudi Arabia to be fully vaccinated with a WHO-approved vaccine and undergo an RT-PCR test 72 h before traveling. Over 20,000 Egyptians performed Ramadan Umrah in April-May 2022 and over 35,000 will perform Hajj in July 2022.

Egypt Ministry of Health and Population (MoHP) started to conduct a yearly survey among Egyptians returning from the Hajj pilgrimage in 2009 as influenza pandemic emerged, for risk assessment and prevention strategy development. The survey was not conducted in 2020–2021 because Umrah and Hajj were suspended.

Recently the WHO Director-General has warned that the pandemic is most certainly not over, and a new and more dangerous variant could emerge at any time where vast numbers of people remain unprotected [8]. With this in mind, MoHP opted to conduct two surveys this year: one among Egyptian pilgrims returning from Ramadan Umrah and the other among Hajj pilgrimage. This report describes results of the survey conducted among Egyptian pilgrims returned from year 2022 Ramadan Umrah, to monitor SARS-CoV-2 and influenza activity and assess the risk of imported acute respiratory virus infections. In addition, whole-genome sequencing of a sample from SARS-CoV2 isolates was performed for timely identifying circulating variants of interest (VOI) and variants of concern (VOC) to help vaccination planning and pandemic control strategies.

### 1.1 Study design

A cross‐sectional survey was conducted among Egyptian pilgrims returning from Ramadan Umrah 2022 season via Cairo airport between 30th April 2022–5 th May 2022.

### 1.2 Sample size

Sample was calculated using epi info7 statistical software [9]. Considering an estimated risk of 10% for contracting COVID-19, at least a sample size of 754 was required to provide a 95% confidence interval with a precision of 0.03 and a design effect of 2 [10]. The sample size was inflated to 904 pilgrims by 20% to compensate for the refusal rate, reduce the standard error, and provide estimates closer to population values. The survey teams were instructed to involve any pilgrim who would like to participate in the survey and get a free RT-PCR test for COVID-19, after consenting. Overall, 1003 Pilgrims expressed willing to participate and enrolled in the survey.

### 1.3 Study population

Of 95,907 Egyptians pilgrims who performed the Umrah in 2022, approximately 20,000 performed Umrah in Ramadan [11]. Pilgrims returning via Cairo airport were opportunistically contacted on their way to the visa area and invited to participate voluntarily in the survey. Subjects were briefed regarding the study objectives and methods. Those who verbally consented were interviewed by MoHP epidemiologists at the airport clinic to protect their privacy and confidentiality. The interview was conducted using a standardized linelist that includes participants’ demographics, clinical information, vaccination status against COVID-19, influenza, and meningitis, type of COVID-19 vaccine, and the date of last dose received. Data was entered into an online application during the interview.

Patients were asked to provide nasopharyngeal and oropharyngeal (NP/OP) swabs for early detection and management of acute respiratory viral infections. Specimens were stored in viral transport media (VTM), preserved in nitrogen tanks, and transported to the Central Public Health Laboratory (CPHL) in Cairo within 24 h for influenza and SARS-CoV-2 testing by Real-Time polymerase chain reaction (RT-PCR). Patients with positive testing results were contacted for follow-up and medical advice.

## 2 Laboratory procedures

### 2.1 Detection of SARS-CoV-2 and influenza A viruses

Samples were collected with flocked nasopharyngeal swabs and immersed in a viral transport medium for delivery to CPHL for testing using SARS-CoV2 PCR protocols. Briefly, RNA was extracted using chemagic 360 equipment (PerkinElmer Inc) was used to extract nucleic acid from the clinical samples. A VIASURE SARS-CoV-2 RT-PCR Detection Kit was used to identify SARS-CoV-2 RNA (ORF1ab and N gene) (Certest Biotec SL, Spain). Positive samples were verified for SARS-CoV-2 using a Cobas 6800 system whereby the RT-PCR runs were done in duplicate and according to the manufacturer's instructions (Roche Holding AG). All specimens were tested by RT-PCR for influenza A and B viruses. Specimens positive for influenza A were further tested for A subtype according to WHO guidelines [12].

## 3 SARS-CoV-2 whole-genome sequencing

All samples selected for whole-genome sequencing had nucleic acid freshly extracted from the original samples. The extracted total RNA was subjected to ribosomal RNA removal using the Ribo-Zero rRNA Removal Kit. All samples were processed using the Illumina COVIDSeq protocol according to the manufacturer's instructions (Illumina, San Diego, CA). The first-strand synthesis was performed using random hexamer primers and followed by two separate multiplex PCR reactions. The pooled PCR amplicons were processed for tagmentation and adapter ligation using IDT for Illumina Nextera UD Indexes. Pooled samples were quantified using the 2.0 fluorometer (Invitrogen Inc., Waltham, MA), then normalized to 4 nM concentration for sequencing on an Illumina MiSeq platform using a MiSeq Reagent Kit v3 (2 × 300 cycles) (Illumina Inc, San Diego, CA, USA).

Genomic analysis and strain typing of SARS-CoV-2 variants is one of the sequence analysis techniques available with the CovidSeq assay. Adequate sequencing requires depth coverage of at least 100 strain-distinguishing regions of the open reading frame 1a (ORF1a), S, N, and ORF8 genes. With the exception of two sequences, all samples produced sufficient strain-typeable sequences, and no sample had a mixed population of viruses. The reads were aligned with the reference genome (NC_045512.2) using CLC Genomics Workbench version 20 (CLC Bio, Qiagen) through workflow. Enrichment analysis was performed for the detected mutations in Umrah samples and compared to 113 random samples from the circulating SARS-Cov2 variants in Egypt obtained from patients presented to the national Egyptian surveillance for SARS-CoV-2 in the same period of time. Pangolin (**https://pangolin.cog-uk.io/**) criteria was used to classify the lineage and clade of the collected samples.

### 3.1 Data analysis

Data was entered using smartphones during the interview. It was extracted in MS Excel format and analyzed using Epi info7. Descriptive data analysis was performed to describe the demographic and epidemiologic characteristics of patients with SARS-CoV-2 and patients with influenza using numbers and frequencies. Rate of SARs-CoV-2 and influenza infections were calculated by dividing number of patients who tested positive for the specific virus divided by the number of study participants. Rates were weighted using the flight number and date of arrival. Vaccination data of COVID-19 positively tested patients were compared to those with negative results to estimate the effectiveness of different types of vaccines, effectiveness of booster dose, and different vaccination regimens for prevention of SARS-CoV-2 infection using chi [2] test, with a significance level of< 0.05. To eliminate confounders, we stratified data by the period since last dose of vaccination.

## 4 Results

Overall, 1003 subjects participated in the survey. Their mean age was 50.9 ± 13 years, 65.9% were between 36 and 56 years of age, 594 (59.2%) were males, 47.8% from Lower Egypt region. Of them 76 (7.6%) were positive for a respiratory virus including 67 (88.2%) positive for SARS-CoV-2, 7 (9.2%) for influenza virus, and 2 (2.6%) for SARS-CoV-2/influenza virus coinfection. The weighted SARS-CoV-2 infection rate was 5.9% and influenza rate was 0.9% (Table 1).

**Table 1:** Characteristics of study subjects and patients by viral cause of acute respiratory infection among the Egyptian pilgrims returning from 2022 Ramadan Umrah.

	All subjects (n = 1003)		Positive for SARS-CoV-2 (n = 67)		Positive for influenza viruses (n = 7)		Negative for both viruses (n = 927)*		P value
Arrival date	No.	%	No.	%	No.	%	No.	%	
30-Apr	24	2.4	4	6.0	0	0.0	20	2.2	0.445
1-May	114	11.4	12	17.9	1	14.3	101	10.9	
2-May	260	25.9	18	26.9	3	42.9	239	25.8	
3-May	235	23.4	16	23.9	1	14.3	217	23.4	
4-May	370	36.9	17	25.4	2	28.6	350	37.8	
Mean age years	50.9 ± 13		49.4 ± 14		55.9 ± 11		50.9 ± 13		0.233
Age groups									
1–14	5	0.5	1	1.5	0	0.0	4	0.4	0.391
15–35	127	12.7	11	16.4	0	0.0	116	12.5	
36–50	287	28.6	16	23.9	2	28.6	269	29.0	
51–65	374	37.3	28	41.8	4	57.1	341	36.8	
> 65	112	11.2	4	6.0	1	14.3	106	11.4	
Missing	98	9.8	7	10.4	0	0.0	91	9.8	
Gender									
Males	595	59.3	38	56.7	3	42.9	552	59.5	0.745
Females	409	40.8	29	43.3	4	57.1	375	40.5	
Region									
Lower Egypt	477	47.6	32	47.8	4	57.1	441	47.6	0.331
Urban governorates	258	25.7	19	28.4	2	28.6	237	25.6	
Upper Egypt	241	24.0	13	19.4	1	14.3	225	24.3	
Frontier	8	0.8	0	0.0	0	0.0	8	0.9	
Missing	17	1.7	2	3.0	0	0.0	15	1.6	
Symptoms									
Mild symptoms	59	5.9	35	52.2	0	0.0	23	2.5	< 0.001
Asymptomatic	941	93.8	32	43.3	7	100.0	904	97.5	
Unable to reach	3	0.3	3	4.5	0	0.0	0	0.0	
Contacted ARI case									
Yes	3	0.3	0	0.0	0	0.0	3	0.3	0.971
No	969	96.6	62	92.5	7	100.0	898	96.9	
Not sure	31	3.1	5	7.5	0	0.0	26	2.8	

* Two patients had SARS-CoV-2/influenza coinfection.

SARS-CoV-2 infected patients were insignificantly younger than those positive for influenza virus infection (49.4 ± 14 vs 55.9 ± 11, p = 0.239), with higher percent of males ( 56.7 vs 42.9%, p = 0.378), more symptomatic (52.2 vs 0.0%, p < 0.001). No difference found between the two groups regarding residence in different demographic areas or difference in the date of arrival, and none of the patients had recently contacted a case with respiratory symptoms (Table 1).

Percent of SARS-CoV-2 infected patients was lowest (3.5%) among those who received inactivated vaccine type and was highest (9.0%) among those who received more than one type. No significant difference identified between receivers of different vaccine types (p = 0.486). In addition, there was no significant difference in infection rate between those who received a booster dose and those who did not, even after stratification by time since the last dose (7.4 vs 6.3%, p = 0.226). The infection rate was insignificantly higher in the first month after vaccination and after 3 months of vaccination (Table 2).

**Table 2:** COVID-19 vaccination status of the Egyptian pilgrims returning from 2022 Ramadan Umrah.

COVID-19 vaccination	All subjects (n = 1003)		Positive for SARS-CoV-2* (n = 67) (Vaccine breakthrough)		Negative for SARS-CoV-2 (n = 934)		P-value
	No.	%	No.	%	No.	%	
Vaccine type							
Viral vector	432	43.1	30	7.0	401	93.0	0.486
mRNA	258	25.7	19	7.4	239	92.6	
Inactivated	142	14.2	5	3.5	137	96.5	
2 types	111	11.1	10	9.0	101	91.0	
DK	60	6.0	3	5.1	56	94.9	
vaccine booster dose							
Yes	367	36.6	27	7.4	339	92.6	0.226
No	636	63.4	40	6.3	595	93.7	
Vaccine regimen in booster dose receivers							
Heterogeneous	101	27.5	10	9.9	91	90.1	0.715
Homogenous	259	70.6	17	6.6	242	93.4	
DK	7	1.9	1	14.3	6	85.7	
Duration since the last dose							
One month	99	9.9	10	10.2	88	89.8	0.324
1–3 months	340	33.9	20	5.9	320	94.1	
> 3 months	377	37.6	28	7.4	348	92.6	
DK	187	18.6	9	4.8	178	95.2	

* The two patients with SARS-CoV-2/influenza coinfection were excluded.

**Fig. 1:**
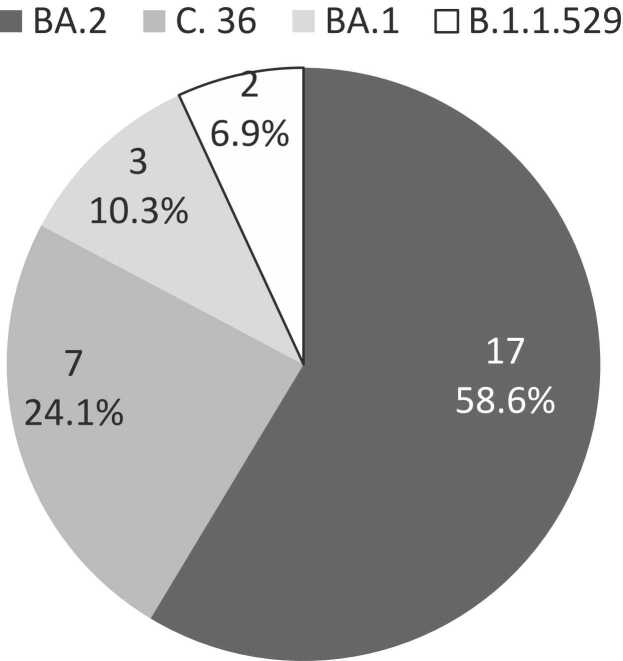
Gene sequencing of 29 SARS-CoV-2 samples collected from the Egyptian pilgrims returning from 2022 Ramadan Umrah.

Among 29 gene sequenced SARS-CoV-2 specimens, 17 (58.6%) were BA.2, 7 (24.1%) C.36, 3 (10.3%) BA.1 and 2 B.1.1.529 (6.9%) Variant (Fig. 1).

Of the 67 patients with SARS-CoV-2, 59 (5.9%) reported having mild acute respiratory symptoms during the Umrah period or one week after returning with no one needed hospitalization (Table 1). Percent of symptomatic patients was highest among patients infected with C. 36 variant (71.4%) and lowest among BA.1 variant infected patients (33.3%).

**Fig. 2:**
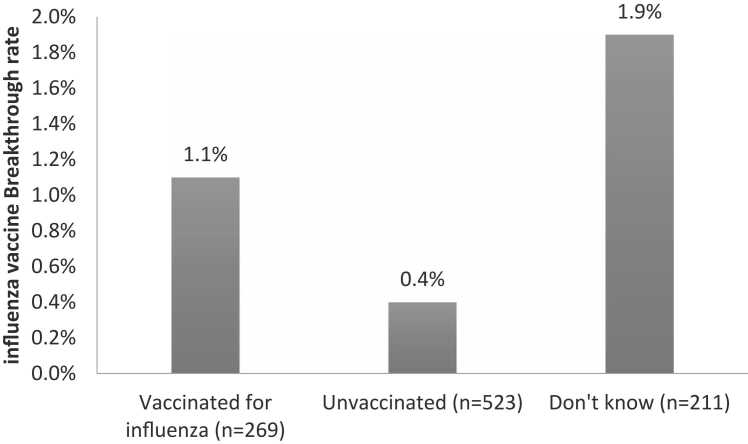
Rate of influenza vaccine breakthrough among the Egyptian pilgrims returning from 2022 Ramadan Umrah.

Of all study subjects, 269 (26.8%) mentioned they had received the influenza vaccine before departure including 3 (1.1%) had influenza viral infection. While among 523 influenza unvaccinated subjects 2 (0.4%) had influenza infection and among 211 subjects with missed influenza vaccination information 4 (1.9%) were infected. The difference between the three groups was insignificant (p = 0.132) (Fig. 2).

Among seven patients with influenza viral infection only, 4 (44.4%) had Flu-B subtype, 3 (33.3%) A/H1, and 2 (22.2%) A/H3. None of the influenza patients had symptoms.

Two patients had SARS-CoV-2/influenza coinfection, a male 77 years of age who had no symptoms, received 2 doses of viral vector vaccine with the last dose in<one month from infection, did not have influenza vaccine, infected with SARS-CoV-2 BA.2 variant and A/H1 subtype. The other patient is a female 55 years of age, had mild symptoms, received 3 doses of COVID-19 vaccine that she does not know its type with the last dose since> 3 months, received influenza vaccine, Influenza subtype was A/H3, and no sequencing performed for SARS-CoV-2. Both patients were from Upper Egypt region.

Whole-gene sequence analysis showed that the overall mutation rate per sample was higher in Umrah survey samples compared to the circulating Egyptian variants. Regarding the type of mutations, the frameshift insertion was present in Umrah survey samples and absent in the Egyptian circulating variants. As per amino acid change, the most frequent events in Umrah survey samples were A63T and Q19E in the M gene, P314L in the NSP12b gene, and Q945Hin S gene. While in the Egyptian circulating variants the most frequent AA changes were A67 frameshift deletion, D614G in the S gene, and the P314L in the NSP12b gene.

## 5 Discussion

This survey study was conducted as a part of the MoHP review of pandemic control measures and vaccination effectiveness plans. We report the incidence, and severity of SARS-CoV-2 and influenza viral infections. In addition to SARS-CoV-2 and influenza vaccines effectiveness in disease prevention, and results of SARS-CoV-2 whole-genome sequencing among Egyptian Ramadan Umrah performers.

Although infection with SARS-CoV-2 may occur among fully vaccinated people, those who have been vaccinated are still much less likely to acquire SARS-CoV-2 or to transmit it to others than those who have not been vaccinated [13]. This study identified a low incidence of SARS-CoV-2 among Egyptian pilgrims compared to other mass gatherings held in the US and Spain that caused much higher rates of COVID-19 transmission among attendees [14], [15].

Although most Umrah seekers are usually old and have chronic conditions, yet most COVID-19 patients in this study were asymptomatic, had mild symptoms with no hospitalizations or death reported. This finding together with the low incidence of COVID-19 could indicate the high effectiveness of vaccines in infection prevention and reducing severity and mortality from COVID-19. Another possible reason could be the low rate of SARS-CoV-2 circulation reported by most countries during this period [6].

Earlier studies reported a higher incidence of COVID-19 among men and patients aged 50 and older, however most of these studies were conducted early in the course of the pandemic [16]. In contrast to other studies, this study found no significant difference between different age groups or genders in the infection rate of SARS-CoV-2. One reason could be the change in SARS-CoV-2 behavior as new variants evolve. Virus behavior should be monitored to inform preventive measures and vaccination policy. The similar SARS-CoV-2 infection rates among all ages and genders could support the recommendation for vaccination of the entire population During this phase of the pandemic.

The study found that no patients with SARS-CoV-2 reported contacting someone with respiratory symptoms. This may highlight the importance of indirect transmission through contact with contaminated surfaces as new variants with higher transmissibility emerge, especially in hot and humid weather. Both cleaning and disinfection are recommended to inactivate SARS-CoV-2 and can reduce the risk of indirect transmission [17].

Currently, Omicron is the dominant variant circulating globally, accounting for nearly all sequences reported to WHO. The proportion of reported sequences designated that BA.2 having been increasing relative to BA.1 [18]. In March 2022, Saudi Arabia reported that 99.8% of all SARS-CoV-2 variants circulating in the Kingdom were Omicron, while Delta accounted for only 0.1%, with the most common descendant lineages reported were BA.1, BA.1.1, BA.2, and BA.3. BA.1 was the most dominant variant and the proportion of BA.2 was increasing since the end of 2021 [4]. Similar findings were reported in this study where Omicron was the only variant found in the sequenced isolates including BA.1, BA.1.1 (or Nextstrain clade 21 K) and BA.2 (or Nextstrain clade 21 L) and C.36 (or Nextstrain clade 20D) sublineages.

C.36 was first detected in Egypt in December 2020 and was almost replaced by BA.1 and BA.2 in 2022 (unpublished data). It was de-escalated as it is no longer detected or detected at extremely low levels [19]. C.36 has no obvious impact on transmissibility or severity, but it has a higher impact on evading vaccine-acquired immunity. The study showed that the C.36 variant had re-emerged among Egyptians returning from the Umrah after exposure to persons from many nations. C.36 variant patients experienced more symptoms than BA.1 and BA.2. This could highlight the possibility of re-emergence of de-escalated variants in time or place and emphasize the importance of closely monitoring SARS-CoV-2 different variants.

Studies have shown that BA.2 -currently the most common sublineage reported- is inherently more transmissible than BA.1. Real-world data from South Africa, the United Kingdom, and Denmark indicated that there is no difference between BA.2 and BA.1 in the transmissibility, clinical severity, and ability to escape vaccines. In addition, both variants are having a relatively low potential for causing severe disease compared to delta variant [18].

Similarly, this study findings did not find a difference between BA.1 and BA.2 in infection severity and both caused mild disease with no hospitalization or deaths. However, these results should be interpreted with caution because the study design may be subject to selection bias and the results are based on relatively small numbers.

Studies showed that vaccine effectiveness after receipt of both 2 and 3 doses was lower during the Omicron-predominant period than during the Delta-predominant period, and that the effectiveness of the booster dose is decreasing over time [20], [21]. In accordance with this, our study did not find a significant difference between having a booster dose or not in the rate of SARS-CoV-2 infection. This finding could support a yearly COVID-19 schedule vaccination rather than a booster dose. More data are needed to assess this finding across studies.

It was suggested that heterologous vaccination regimen is an opportunity to increase vaccination programs’ flexibility in response to the fluctuations in supply, especially for countries with scarce vaccine access and when different vaccines are available at different times. It was also suggested that heterologous regimens have the potential to produce a stronger response and are necessary with the emergence of new variants [22], [23]. However, recently it was found that three-dose mRNA regimen is more effective than a heterologous regimen against COVID-19 infections using two dose adenovirus vector vaccines with one mRNA vaccine [24]. Our study found no significant difference between homologous and heterologous vaccination regimens in the prevention of symptomatic and asymptomatic infection. However, these results should be interpreted with caution as confounders may exist and could bias analysis.

Although influenza vaccination is required by Egypt health authorities for all Egyptian Hajj and Umrah pilgrims prior to departure, yet 2012–2015 surveys revealed that only 19.7% of the Egyptian pilgrims have been vaccinated for influenza [25].

This study found a higher percentage of influenza vaccinees among Egyptian pilgrims. The improvement in influenza vaccination rate is probably caused by the efforts made to overcome vaccine hesitancy in Egypt for the COVID-19 vaccine.

The study also found a lower influenza infection rate among returnees from Umrah compared to 2012–2105 surveys [25]. This could reflect the predominance of SARS-CoV-2 over the influenza virus during the ongoing COVID-19 pandemic rather than influenza vaccine effectiveness as the study did not find a significant difference between the vaccinated and unvaccinated regarding influenza infection rate.

The finding that Flu-B represents a significant proportion of influenza cases necessitates the revision of influenza vaccine type and formulation with continued isolate sharing to enhance influenza vaccine effectiveness and prevent future influenza pandemics.

Two asymptomatic patients with SARS-CoV-2/influenza coinfection were identified among pilgrims. Co-infection in patients with mild symptoms or asymptomatic was previously reported in Egypt [26]. Both were vaccinated for SARS-CoV-2 and unvaccinated for influenza. This could reflect the usefulness of COVID-19 vaccines in preventing severe disease, hospitalization, and deaths and highlights the need to enforce influenza vaccination before Hajj and Umrah for Egyptian pilgrims.

The whole-genome sequencing indicated that more frequent mutations occurred in the pilgrims’ SARS-CoV-2 isolates than the virus circulating in Egypt at the same time. This highlights the importance of maintaining virology surveillance, especially among returning travelers for early detection of new variants of concern. Studies recommended the implementation of surveillance among travelers near points of entry to identify patients infected with lineages of interest and provide information on virus diversity leading to better preparedness and response to newly emerging SARS-CoV-2 variants [27].

## 6 Conclusions

A cross-sectional survey was conducted among Egyptian pilgrims returning from Ramadan Umrah 2022 the second largest Islamic mass gathering to prepare for the Hajj large mass gathering that will be held in July 2022. The survey indicated a low rate of infection with SARs-CoV-2 among pilgrims. All patients had mild symptoms or were asymptomatic with no hospitalization or deaths reported. This could indicate the value of vaccination in limiting infection, severe disease, hospitalization, and deaths and highlight the need to enforce COVID-19 vaccination before large mass gatherings. Omicron sublanguage BA.2 is found the predominant variant with low potential for causing severe disease yet with a higher ability to escape vaccine. Together with the re-emergence of C.36 sublineage, monitoring SARS-CoV-2 genome sequencing is essential, especially among travelers to tailor vaccination strategy and preventive measures.

The study indicated that all types of COVID-19 vaccines are equally effective, while no evidence found on the effectiveness of the administration of a booster dose over full vaccination or of the heterologous vaccination regimens over homologous in the prevention of infection.

The study indicated better influenza vaccination coverage and lower influenza infection rate compared to previous seasons; however, this could be related to the predominance of SARS-CoV-2 over the influenza virus during the pandemic. Enhancing influenza vaccination with monitoring of influenza activity and sharing isolates is essential to prepare for future expected influenza epidemics and pandemics.

## Funding statement

This research received no specific grant from any funding agency in the public, commercial, or not-for-profit sectors.

## Ethics approval statement

the study was approved by the Ministry of Health and Population Ethics committee and institutional review board (IRB).

## Patient consent statement

All subjects verbally consented to participate after they were fully informed about the aims of the survey and the risks associated. Interviews were conducted by MoHP epidemiologists in a separate location in the airport clinic, and anonymity was maintained for preserving participants' confidentiality.

## Permission to reproduce material from other sources

Permission is not required as no materials from other sources were used in the manuscript.

## Conflict of interest disclosure

The authors have no conflicts of interest to declare. All co-authors have seen and agree with the contents of the manuscript and there is no financial interest to report. We certify that the submission is original work and is not under review at any other publication.

## Data Availability

The datasets generated and/or analyzed during the current study are not publicly available due to privacy restrictions but are available from the Egypt Ministry of Health and Population upon reasonable request.
